# Food for our future: the nutritional science behind the sustainable fungal protein – mycoprotein. A symposium review

**DOI:** 10.1017/jns.2023.29

**Published:** 2023-04-11

**Authors:** Emma J. Derbyshire, Hannah Theobald, Benjamin T Wall, Francis Stephens

**Affiliations:** 1 Nutritional Insight, Surrey, UK; 2 Marlow Foods, Stokesley, UK; 3 University of Exeter, Exeter, UK

**Keywords:** Future nutrition, Fungal protein, Health, Mycoprotein, Sustainability

## Abstract

Mycoprotein is a well-established and sustainably produced, protein-rich, high-fibre, whole food source derived from the fermentation of fungus. The present publication is based on a symposium held during the Nutrition Society Summer Conference 2022 in Sheffield that explored ‘*Food for our Future: The Science Behind Sustainable Fungal Proteins*’. A growing body of science links mycoprotein consumption with muscle/myofibrillar protein synthesis and improved cardiometabolic (principally lipid) markers. As described at this event, given the accumulating health and sustainability credentials of mycoprotein, there is great scope for fungal-derived mycoprotein to sit more prominently within future, updated food-based dietary guidelines.

## Introduction

The ‘*Food and Nutrition: Pathways to a Sustainable Future*’ conference was undertaken at Sheffield University in July 2022 with a symposium dedicated to ‘*The Science Behind Sustainable Fungal Proteins*’. The present publication collates evidence presented at this symposium. The need for sustainable food proteins is becoming a focal point of interest for several reasons. Firstly, the global population is enlarging – by the year 2050, this is predicted to exceed 10 billion people^(1)^. Alongside this, the daily per capita supply of protein has risen globally from 61 g in 1961 to 81 g in 2013 showing that individual daily protein intakes have been rising. The expanding global population coupled with changing socio-demographics and reliance on food proteins with environmental ramifications (high greenhouse gas emissions, water and land use) is creating a ‘perfect storm’ when it comes to securing future protein supplies^(2)^. For these reasons, alternative proteins provide a solution to meet growing protein demands within environmental limits^(3)^ and mycoprotein is one of these.

The present publication focuses on topics relevant to foods for our future and the significance of sustainable fungal proteins. We discuss the body of science in the field and latest research findings presented at the symposium. The aim of the symposium was to broaden awareness of fungal food proteins. Focus was given on mycoprotein and its role(s) in addressing global food sustainability challenges, as a bioavailable protein source to support muscle protein synthesis (MPS), resistance-training induced muscle adaptations, effects on cardiometabolic health and its potential place within food-based dietary guidelines (FBDG).

## History and nutritional composition of mycoprotein

Mycoprotein is a well-established and sustainably produced, protein-rich, high-fibre, whole food source, derived from the fermentation of fungus. Mycoprotein is derived from the *Fusarium venenatum* fungus and is the main component of Quorn™ foods which was launched in 1985^(4)^ and is now sold in twenty different countries. *F. venenatum* A3/5 falls under the phylum of Ascomycota and is classed as a true fungus^(5)^. This filamentous micro fungus was first found in soil samples from a field located in Marlow, Buckinghamshire in the 1960s^(6)^. By the late 1960s, *F. venenatum* was used to develop mycoprotein and by 1984 was authorised for sale as a food by the UK Ministry of Agriculture, Fisheries and Food^(7)^.

Today, mycoprotein is produced vertically in fermenters^(8)^. Harrison and Johnson^(9)^ have published a useful overview of mycoprotein production. Salts, pure glucose and ammonia are fed in through an airline and small amounts of biotin and choline added to facilitate growth. The fermenters operate continuously, running for approximately a month at a time. The fermentation broth is heat treated as they leave the fermenter to reduce the ribonucleic acid (RNA) content. Next, the product is pasteurised, and centrifuged to remove surplus liquid, leaving a paste resembling a dough. The paste is heated to set the dough into a solid billet, which is then cooled, cut into shapes, or minced and then frozen. The freezing processed enables ice crystals to form which forms hyphae into bundles that closely resemble the fibres seen in chicken. A new study^(10)^ mapping the functionality of mycoprotein throughout the fermentation process concluded that there were previously unreported gelling, foaming and/or emulsifying properties for the different Quorn™ streams that could be further used to produce additional novel products.

From a nutritional perspective, mycoprotein has a low energy density, is low in total and saturated fat and contains negligible amounts of cholesterol^(11)^. It provides the nine main essential amino acids (AAs) and has a protein digestibility-corrected amino acid score of 0⋅996, indicating that it is a high-quality protein^(12)^. It also meets the UK and European Commission^(13,14)^ nutrition claims requirements to be classed as ‘high in fibre’ due to it providing at least 6 g of fibre per 100 g. Mycoprotein is, therefore, a high-fibre food source and typically one-third of chitin (poly *n*-acetyl glucosamine) and two-thirds β-glucan (both 1,3 and 1,6)^(15)^. Given the fibre profile of mycoprotein, as part of the Food and Drink Federations (FDF) new ‘Action on Fibre’ campaign^(16)^, Quorn™ have pledged to help narrow the gap between fibre intakes and dietary recommendations by: (1) making higher fibre diets more appealing, normal and easy for the population, (2) working with partners to raise awareness, resources and advocacy and (3) expanding their scientific research programme.

Mycoprotein further meets the UK and European Commission^(13,14)^ conditions of use for riboflavin, folate, source claims and high in folate, phosphorus, zinc and manganese claims, providing at least 30 % of the Nutrient Reference Value. Mycoprotein also provides useful amounts of choline (180 mg/100 g) but cannot be considered a source as no Nutrient Reference Value (NRV) has been set by Public Health England, UK or the European Food Safety Authority. Certain fungi such as mushrooms can contribute to 5-A-Day providing that this is an 80-gram portion – equivalent to 14 button or 3–4 heaped tablespoons of mushrooms, or 2 tablespoons of dried mushrooms^(17)^. However, mycoprotein derived from fungi is not currently recognised as contributing to the 5-A-Day.

Mycoprotein is not yet included pictorially within the Eatwell guide infographic though it is mentioned in the Eatwell guide booklet where it states under the closer look at protein section that ‘*Other vegetable-based sources of protein include tofu, bean curd and mycoprotein; all of which are widely available in most retailers*’. Given the growing evidence-based reflection is needed to consider its integration. The British Dietetic Association One Blue Dot Report^(18)^ which makes environmentally sustainable diet recommendations for the UK advises that daily intakes of plant-proteins should be raised, and we should ‘*Prioritise beans and lentils, soya (beans, mince, nuts, tofu), mycoprotein (Quorn™), nuts and seeds*’. A recent survey^(19)^ of 188 dietitians identified that 72 % agreed that UK EatWell guidance should be updated to integrate sustainable diets with 92 % reporting that meat-alternatives should be represented pictorially and 82 % confirming that mycoprotein should be represented within such an infographic.

## Mycoprotein bioavailability and resistance-training muscle adaptations

Mycoprotein has been shown to be a bioavailable protein source^(20)^. Dietary proteins comprise AAs linked by peptide bonds that are hydrolysed in the lumen of the gastrointestinal tract to form dipeptides, and tripeptides which are then utilised by bacteria in the small intestine or absorbed into enterocytes^(21)^. The AAs that are not degraded in the small intestine move into the portal vein and peripheral circulation for protein synthesis in skeletal muscle and other tissues^(21)^. Several anabolic stimuli can stimulate MPS which includes nutrition (protein ingestion), daily physical activity and resistance exercise (RE)^(22)^.

There are different speeds of protein turnover across pools in the body and AA availability is an important regulator of muscle protein metabolism. Rates of muscle protein turnover in humans are around 300–400 g/d – amounts that largely exceed levels of protein intake (≈50–80 g/d)^(23)^. After undertaking RE the response of MPS lasts for approximately 24–48 h^(24)^. Therefore, interactions between any meals ingested and protein anabolism will occur during this time and impact on levels of muscle hypertrophy and reconditioning^(24)^. Leucine is a particularly important branched-chain AA, responsible for switching on intracellular machinery^(25)^. It enhances protein synthesis via the activation of the mammalian target of rapamycin (mTOR) signalling pathway in skeletal muscle^(25)^. Around 80 % of leucine is typically used for MPS and rest is converted to metabolites (α-ketoisocaproate and β-hydroxy-β-methylbutyrate) in skeletal muscle^(25)^. Subsequently, it is thought that some of the main functions of leucine, i.e. protein synthesis may be modulated by these metabolites. AAs act as a signal to muscle tissue depending on the size and type of meal. The foods that we ingest, and corresponding levels of AAs oscillate throughout the day.

Dietary protein is a central macronutrient but the concept of what constitutes ‘protein quality’ is complex. This can encompass: (1) the quantity of AAs (protein density), (2) digestibility and (3) bioavailability. Recent research has studied whether bolus ingestion of mycoprotein (as part of its wholefood matrix) would stimulate MPS rates more than a leucine-matched bolus of protein concentrated from mycoprotein. It has been shown that protein consumption significantly raised plasma essential amino acid and leucine concentrations (*P* < 0⋅0001), but more rapidly and to greater magnitudes when derived from the milk protein concentrate^(26)^. MPS synthesis, however, increased in rested and exercised muscle in both conditions indicating that the ‘form’ of delivery was irrespective.

Taken together, there has been growing interest in how much protein we need^(27,28)^, the daily distribution of protein^(29,30)^ and now the ‘type’ of protein is gaining attention from a health and environmental perspective^(31)^. To draw to an end, a recent randomised-controlled trial^(32)^ determined whether a mycoprotein-based diet could support daily myofibrillar protein synthesis rates that were comparable with an omnivorous diet. The study recruited older adults (*n* 19; mean age 66 years) who ingested a 3-day isocaloric high-protein diet where protein was derived from animal or mycoprotein sources. They also undertook a daily unilateral leg extension RE. The research team found that both dietary protein sources resulted in equivalent rested and exercised daily myofibrillar protein synthesis rates. Overall, this implies that mycoprotein can robustly increase MPS rates, sustaining protein synthesis rates to levels comparable to omnivorous diets over several days.

## Mycoprotein and cardiometabolic health

Several studies in the past have shown beneficial effects on glycaemia and insulinaemia when mycoprotein has been ingested acutely^(33–35)^. Turnbull and Ward^(33)^ was one of the first publications to demonstrate that mycoprotein (ingested in milkshake) could significantly improve postprandial glycaemic response 60 min after ingestion (13 % reduction) and insulinaemic response 30 min after consumption (19 % reduction) in healthy young males and females.

Earlier work by the Imperial College research^(36)^ group undertook randomised controlled trials showing that mycoprotein test meals (low 44 g; medium 88 g or high 132 g) reduced insulin release at all levels of intake in overweight young adults compared with a chicken meal control. Mechanisms were investigated and not thought to involve changes in the satiety hormones, peptide tyrosine-tyrosine or glucagon-like peptide-1.

Researchers at the University of Exeter (2021)^(37)^ have further studied the effects of integrating mycoprotein within diets and impacts on insulin sensitivity, glycaemic control and plasma lipoprotein profile. Earlier studies have shown links with mycoprotein ingestion and 0⋅1–0⋅2 mmol reductions in cholesterol across a period of a week^(34,35)^. In a randomised, parallel-group trial^(37)^ normolcholesterolaemic young adults (mean age 24 years) ate a fully controlled diet for 1 week providing 180 g mycoprotein per day or meat/fish. In the mycoprotein group, glycaemic control did not change but free cholesterol, total plasma cholesterol (0⋅4 mmol/l reduction over 1 week), LDL-cholesterol, HDL2-cholesterol and the smaller lipoprotein particles significantly reduced (by 14–19 %) compared with 3–11 % reductions in the control. The mycoprotein diet provided 6 g/d more dietary fibre^(37)^ proposed to induce cholesterol-lowering effects; a theory that is in line with epidemiological and other intervention studies^(38,39)^. The type of fibre present in mycoprotein – two-thirds branched β-glucan and one-third chitin which creates a fibrous 88 % insoluble matrix may also be attributed to these cholesterol-lowering mechanisms^(34,35)^.

When considering such mechanisms relating to dietary fibre intake and cardiometabolic health, it is important to consider which fibre components possess the physical characteristics required to induce favourable health effects^(40)^. It is well recognised that mycoprotein is a high-fibre food source. The fibre form is typically one-third of chitin (poly *n*-acetyl glucosamine) and two-thirds β-glucan (both 1,3 and 1,6)^(15)^. It is known that fibre fermentation yields short-chain fatty acids (SCFAs), induced by colonic bacteria which can modulate glucose and lipid parameters^(41)^. This, in turn, has been associated with satiety and reduced energy intake^(42)^. Research at the University of Glasgow^(43)^ has studied the SCFA-generating capacity of mycoprotein. An analysis of *in vitro* batch fermentations demonstrated that both mycoprotein and mycoprotein fibre were both fermentable and produced a total SCFA production of 24⋅9 (1⋅7) and 61⋅2 (15⋅7) mmol/l, respectively. An *in vitro* gut model at the Quadram Institute, Norwich Research Park^(44)^ has further studied the mechanisms behind mycoproteins’ ability to modulate blood lipid levels. The study showed that mycoprotein can inhibit gut lipases and sequester bile salts, which could be plausible underpinning mechanisms. Further studies are warranted to add to these findings.

Taken together, these findings show that mycoprotein appears to lower glycaemic and insulinaemic response to a meal. Short-term research^(37)^ indicates that replacing meat with mycoprotein for one week does not affect insulin sensitivity or glycaemic control. However, longer-term research^(34)^ shows that >80 g/d mycoprotein (5 g/d fibre) over 1–8 weeks reduces total and LDL-cholesterol under eucaloric conditions, which may be beneficial for glycaemic control if maintained. Effects appear to be attributed to fibre composition and modified lipid absorption within the gut^(34)^. An array of mechanisms have been purported and largely attributed to the fibre component of mycoprotein. These include the modulation of SCFAs, inhibition of gut lipases and impaired cholesterol/bile absorption. Ongoing research is needed but mycoprotein appears to show great promise as a dietary component for metabolic health.

## Mycoprotein, food sustainability challenges and representation within FBDG

The global food system makes a significant contribution to greenhouse gas emissions (GHGEs), from production through to processing. The message to strive towards the consumption of healthy and balanced diets needs to be sustained, yet the concept of what these constitute is changing as the environmental ramifications of food production methods gains attention. The United Nations Food and Agricultural Organisation^(45)^ defines a sustainable food system as: ‘*a food system that delivers food security and nutrition for all in such a way that the economic, social and environmental bases to generate food security and nutrition for future generations are not compromised*’. An in-depth publication by Bené *et al.*^(46)^ has further identified twenty-seven key indicators as proxies for the sustainability of food systems and within these the role of diet diversification is recognised.

The global population is expanding and anticipated to exceed 10 billion by 2050^(47)^. There are several driving forces behind this which include more females surviving to reproductive age, improved fertility rates and ageing populations^(48)^. In the United Kingdom (UK), the Office for National Statistics^(49)^ project that population is expected to rise by 3⋅2 % from mid-2020 to mid-2030. By the year 2040, this is projected to reach 70 million and the number of people aged 85 years is predicted to double to 3⋅1 million by 2045 (from 1⋅7 million in 2020), forming 4⋅3 % of the UK population^(49)^.

Given these prominent shifts in population growth several food sustainability challenges exist. For example, 2 billion individuals globally are overweight and at the other end of the spectrum around 2 billion have ‘hidden hunger’^(50)^. Animal diseases have disrupted regional and international trade of animal products, competition of land, energy and water is rising and climate change (drought, storms and increasing extreme weather events) have been impacting on food production^(50)^.

A recent publication in *Nature* journal^(51)^ calculated the projected environmental benefits associated with replacing ruminant meat with mycoprotein. It modelled the outcomes of 20, 50 and 80 % replacement of the per-capita protein consumption from ruminant meat with mycoprotein^(51)^. Results showed that 20 % replacement of ruminant meat with mycoprotein globally offsets projected increases in pasture area and lowered annual deforestation and related CO_2_ emissions by half, while also reducing methane emissions^(51)^. It was further concluded that such substitutions would help ruminant meat demand from 2025 to remain static, offsetting projected increases in global pasture demands for feed^(51)^.

While dietary movements have a valuable role to play in facilitating the ease of future food supplies and counteracting environmental ramifications, these changes will not come about unless populations are educated and well-informed about food systems and dietary guidelines. A modelling study^(52)^ using data from FBDG from eighty-five countries demonstrated that most (up to 87 % of countries) were incompatible with the Paris Climate Agreement and other environmental targets. Another review of forty-three national FBDG^(53)^ further revealed that environmental impacts of the diet were considered infrequently, particularly in older sets of FBDG which overlooked and aligned less well with environmental and sociocultural aspects of food and diet. Focusing in on protein messages, a global review of FBDG from ninety countries^(54)^ showed that not all protein messages were universally echoed. Within these, the embedment of alternative fungal-derived proteins, including mycoprotein, are barely mentioned. While sizeable discrepancies between FBDG (and protein guidance within these) exists globally, the potential benefits for the planet from an environmental perspective remains comparatively small.^(55)^

A recent review^(56)^ further argues that current definitions of what constitute ‘protein quality’ are antiquated and should move to include health and environmental outcomes corresponding to specific food protein sources. Indeed, the concept of ‘protein diversification’ is gaining attention and fungal proteins such as mycoprotein have a viable role to play within this^(55,57,58)^. As we have seen in this symposium, research of the past and present shows that mycoprotein has both health and sustainability credentials. At least sixteen controlled trials have now investigated inter-relationships between mycoprotein and health^(11)^. Further work^(59)^ shows that given growing multimorbidity's, expanding and ageing populations, the integration of fungal mycoprotein within daily diets could benefit health across the lifespan, including the narrowing of the present fibre gap.

## Conclusions

The future potential for fungal-derived proteins, including mycoprotein is vast. The symposium described how an established body of science exists, showing that mycoprotein offers great promise as a dietary component that can sustain protein synthesis rates like omnivorous diets^(32)^ and reinforce metabolic health^(34–36)^. Fungi have long been categorised as a separate ‘Third Kingdom’ due to their distinct cellular organisation, with these falling outside the dichotomy of animals and vegetables^(60,61)^. There is, therefore, great scope to embed these more firmly within FBDG as a prominent category^(55)^. It is clear that the movement towards ‘protein diversification’ is becoming increasingly crucial as global populations continue to grow and food production methods become more challenging.
